# Research Trends and Insights Regarding MicroRNAs in Psoriasis: A Comprehensive Analysis

**DOI:** 10.7759/cureus.100071

**Published:** 2025-12-25

**Authors:** Ismail Örs, Nur Cihan Cosansu, Berna Solak

**Affiliations:** 1 Dermatology, Meram State Hospital, Konya, TUR; 2 Dermatology, Sakarya University Training and Research Hospital, Sakarya, TUR; 3 Dermatology, Faculty of Medicine, Sakarya University, Sakarya, TUR

**Keywords:** biomarkers, inflammation, keratinocytes, micrornas, mirna, psoriasis

## Abstract

MicroRNAs (miRNAs) have emerged as key regulators in psoriasis, yet the global research landscape has not been systematically mapped from an informatics and translational perspective. We searched the Web of Science Core Collection on April 23, 2024, for English-language articles and reviews using "TS=(psoriasis) AND TS=(miRNA OR miR)". Records and cited references were exported as TXT/BIB. Bibliometric and network analyses were performed with VOSviewer (v1.6.18, Leiden University, Leiden, Netherlands) and the bibliometrix package in R (v4.2.1, R Foundation for Statistical Computing, Vienna, Austria). Co-authorship, co-occurrence, and citation networks were constructed with predefined thresholds; local vs. global citations were distinguished. Protein-protein/miRNA-target interactions were summarized via STRING (version/date aligned with the bibliometric window). We identified 449 publications (393 articles, 56 reviews) from 2003 to 2024, with a 13.8% annual growth and 28.5 citations per document. Thematic mapping recovered basic themes (psoriasis, keratinocytes, inflammation), motor themes (miR-146a-5p, exosomes, GAB1), and emerging terms (STAT3, adalimumab). A STRING-based summary highlighted hubs (STAT3, PTEN, TRAF6) converging on inflammatory and proliferative pathways. Translational tags co-localized “biomarker/monitoring” with core psoriasis/miRNA clusters, and drug tokens (e.g., adalimumab) appeared in emerging/motor zones, indicating a literature focus that is potentially useful for panel scoping and exploratory endpoint selection. Research on miRNAs in psoriasis shows sustained growth and a shift toward translational topics. The evidence map and prioritized miRNA hubs can inform biomarker panel selection, trial design, and clinical decision support in psoriasis.

## Introduction and background

Psoriasis is a common, chronic inflammatory skin disease with a complex and incompletely understood pathogenesis. It is considered a polygenic and multifactorial disorder, shaped by immune dysregulation and the interplay between genetic susceptibility and environmental factors [1]. A deeper understanding of its molecular underpinnings may contribute to the development of personalized treatment strategies.

MicroRNAs (miRNAs) are small non-coding RNAs (~22 nucleotides) that regulate gene expression post-transcriptionally, primarily by binding to the 3′ untranslated regions of messenger RNAs and inhibiting their translation. They are estimated to influence the expression of over 60% of human protein-coding genes [2]. The discovery of miRNAs has shed light on previously elusive aspects of gene regulation. In the context of psoriasis, miRNAs play critical roles in modulating key cellular processes such as proliferation, apoptosis, and inflammation, all of which are central to disease pathogenesis [3-5]. Consequently, miRNAs have emerged as potential diagnostic biomarkers and therapeutic targets in psoriasis.

Despite the rapidly expanding body of literature, the overall research landscape of miRNAs in psoriasis has not yet been systematically mapped. A comprehensive scientometric approach can clarify how the field has evolved across thematic domains, collaborations, and translational directions. By integrating bibliometric mapping with protein-protein and miRNA-target interaction analyses, this study delineates the key research trends, influential contributors, and emerging molecular hubs in psoriasis-related miRNA research. The resulting evidence framework aims to identify knowledge gaps and guide future investigations toward biomarker development, mechanistic insight, and clinical translation.

## Review

Methods

Search Strategy and Data Source

In this study, the online literature search was conducted on April 23, 2024, using the Web of Science (WoS) Core Collection (Clarivate Analytics, Philadelphia, PA) database, covering the period from 1975 to 2024. The titles, document types, publication years, authors, institutions, keywords, journals, countries, citations, and other data for each document were exported as .txt and .bib files from the full records of all results using the Results Analysis and Citation Report tools of the WoS database. The search included the following indexes within the WoS Core Collection: Science Citation Index Expanded (SCI-EXPANDED), Social Sciences Citation Index (SSCI), Arts & Humanities Citation Index (A&HCI), and Emerging Sources Citation Index (ESCI). The Web of Science Core Collection was selected as the exclusive data source because it offers the most comprehensive and standardized citation indexing, which is essential for the reliability of VOSviewer (Leiden University, Leiden, Netherlands) co-citation and network analyses.

In the search, the following keywords were used, combined with Boolean operators: "TS= "Psoriasis" AND ("miRNA" OR "miR")". The retrieval time for articles meeting the criteria ranged from January 2003 to April 2024. Only articles and review articles were included in the study. Articles written in languages other than English and those classified as early access were excluded.

Data Extraction and Visualized Analysis

Bibliometric analysis and visualization were conducted using VOSviewer (version 1.6.18), STRING database (version 12.0; accessed: April 2024), and the bibliometrix package in R (version 4.2.1, R Foundation for Statistical Computing, Vienna, Austria) [6]. For the interaction network, validated targets were defined as interactions supported by experimental evidence with a minimum confidence score of 0.400 (medium confidence). Network maps were constructed using fractional counting and normalized via the association strength method. A minimum occurrence threshold of five was applied for keywords to ensure interpretability, and synonyms were harmonized using a standard thesaurus file. These software tools were used for creating bibliometric networks and maps, analyzing collaboration networks among authors, and visualizing the co-occurrence of keywords. This study, which does not require ethical approval, was conducted in accordance with the 2013 Declaration of Helsinki.

Since this study focuses on bibliometric indicators and research mapping, inferential statistical testing (e.g., P-values, regressions) and meta-analysis of clinical outcomes were not performed. Consequently, a traditional risk-of-bias assessment for individual studies was not applicable.

Results

Publication and Trend Analysis

**Figure 1:**
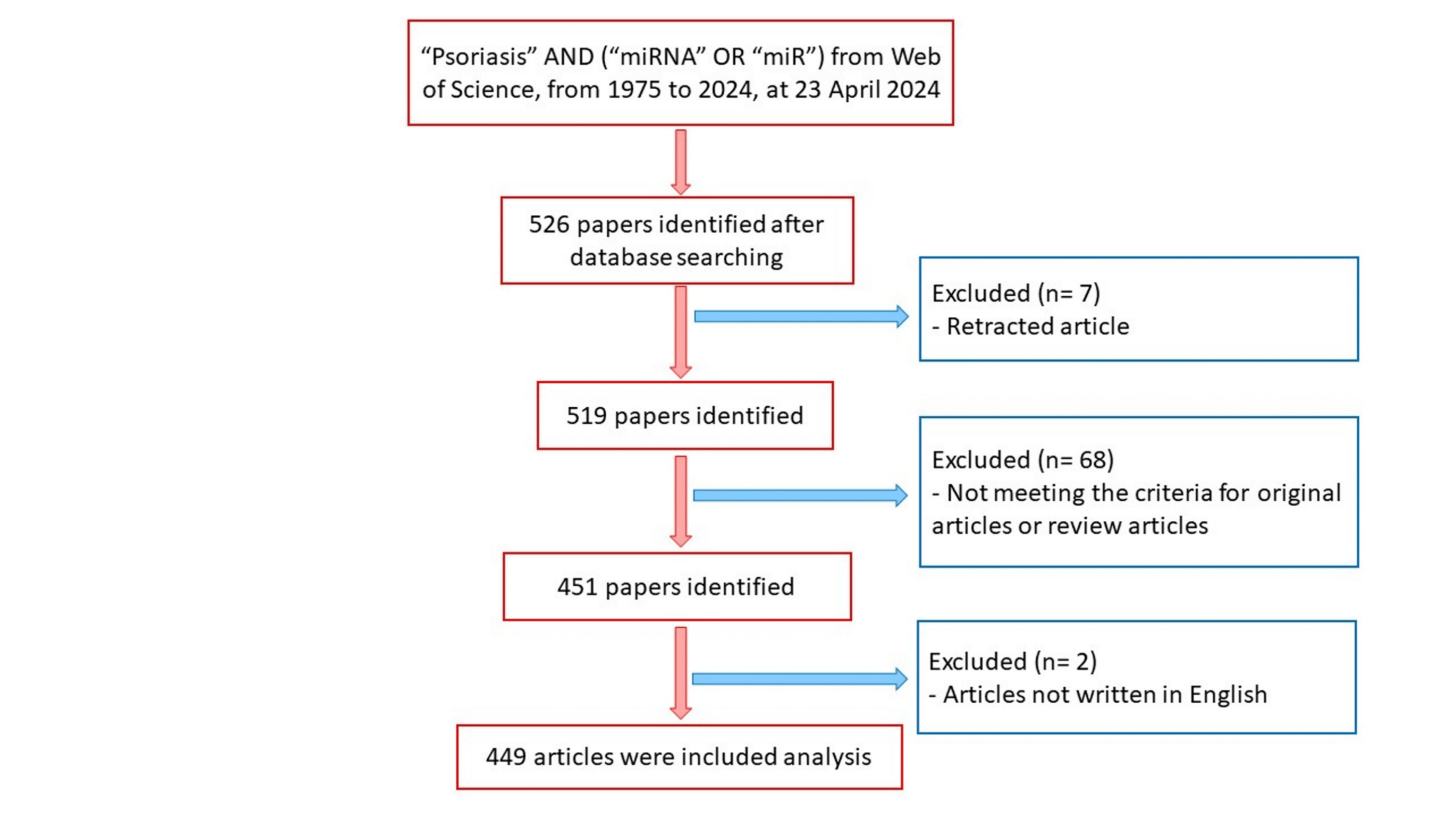
Flowchart of the study.

The search conducted in the Web of Science Core Collection using the specified keywords between 1975 and 2024 initially retrieved 526 publications. Seven retracted articles were excluded, leaving 519 publications. After removing document types other than original articles and review articles, 451 publications remained. Further restriction to those published in English between 2003 and 2024 resulted in 449 publications, comprising 393 (87.5%) original articles and 56 (12.5%) reviews (Figure 1). General information about the dataset is presented in Table 1.

**Table 1:** General information about the data.

Description	Results
Main information about the data	
Timespan	2003-2024
Sources (journals, books, etc.)	222
Documents	449
Annual growth rate	13.8%
Document average age	5.6
Average citation (per document)	28.5
References	15165
Document contents	
Keywords Plus (ID)	1112
Author's keywords (DE)	1067
Authors	
Authors	2448
Authors of single-authored docs	5
Authors collaboration	
Single-authored documents	5
Co-authors (per document)	7.9
International co-authorships rate	17.6%
Document types	
Article	393
Review	56

**Figure 2:**
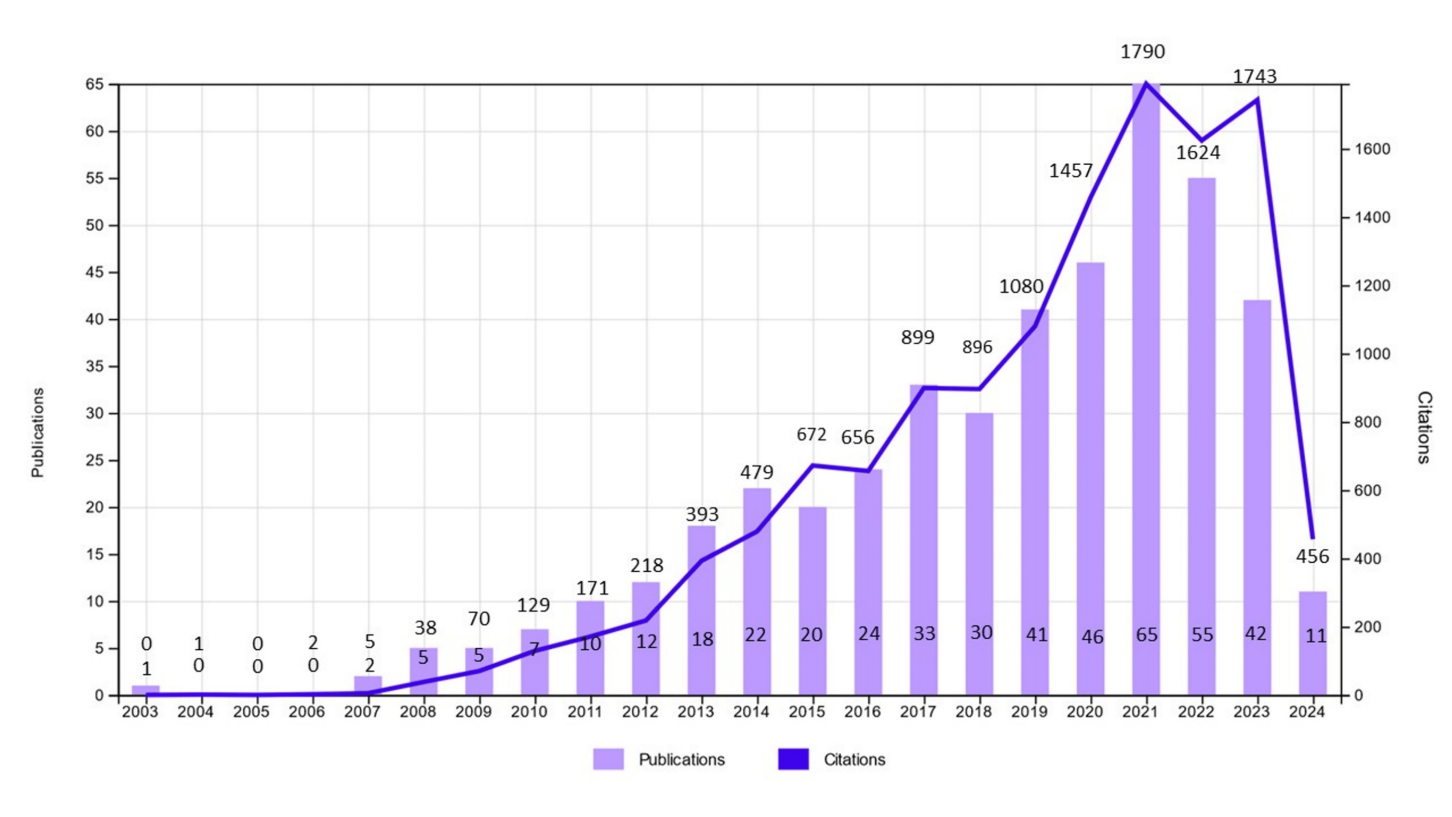
Annual publication and citation trends for microRNA (miRNA) research in psoriasis from 2003 to 2024. The vertical bars represent the number of publications per year, while the line indicates the corresponding annual citation count.

The annual growth rate of the number of papers was 13.8%, with an average of 28.5 citations per document. The annual publication and citation counts are shown in Figure 2.

The annual publication curve demonstrates a rapid increase in annual publications and citations since 2007, culminating in a peak of 65 publications and 1790 citations in 2021. However, a slight decline was noted starting in 2022 and continuing into 2023. As of March 20, 2024, 11 publications have been recorded for the first approximately four months of the year.

Authorship Analysis

The total number of authors in this database was 2,448. The authors with the highest number of papers on this topic are, in order, Li J (26 articles), Li X (22), Pivarcsi A (22), Sonkoly E (21), and Stahle M (20). The authors with the highest total local citation counts are Pivarcsi A (433 citations), Sonkoly E (433), and Stahle M (415), respectively.

Of the authors, 1967 (80.4%) have only one study on this topic, while 271 authors (11.1%) have two studies each. The number of single-authored documents was five (1.1% of all documents). The rate of international co-authorships was 17.6%. Regarding authors' collaboration, the average number of co-authors per document was 7.9.

Analysis of authors' production over time indicated that Pivarcsi A, Sonkoly E, and Stahle M have maintained relatively consistent publication and citation output since 2007. In contrast, in more recent years, Li J, Li X, Wang H, and Wang Y have become increasingly prominent. Clustering by coupling, determined based on citation relationships among authors, together with collaboration network metrics ranked by PageRank, revealed that the authors with the greatest overall importance and influence within the network were Li X, Stahle M, Pivarcsi A, Sonkoly E, and Li J.

Countries and Institutions Analysis

The analyzed publications originated from 51 countries and 887 institutions. The People’s Republic of China ranked first with 199 publications (44.3%), followed by the USA with 52 papers (11.6%) and Sweden with 30 publications (6.7%).

**Figure 3:**
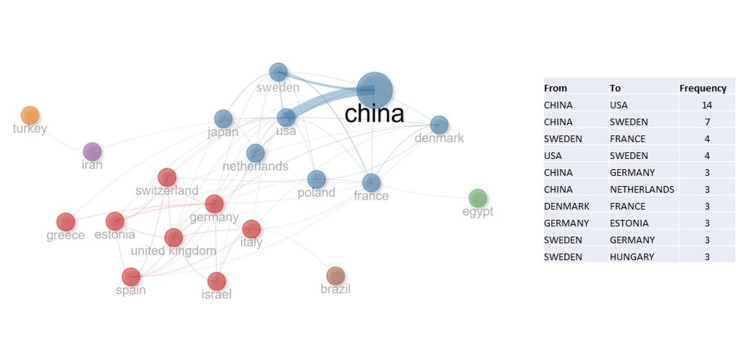
Collaboration network of countries with the highest number of publications in the field. The strongest international collaboration was observed between China and the USA, followed by China and Sweden.

Figure 3 shows the collaboration network of the countries and the frequency of their co-authorships.

The People's Republic of China and the USA demonstrated the most active collaboration with 14 articles. The second most frequent collaboration is between the People's Republic of China and Sweden, with seven articles.

**Figure 4:**
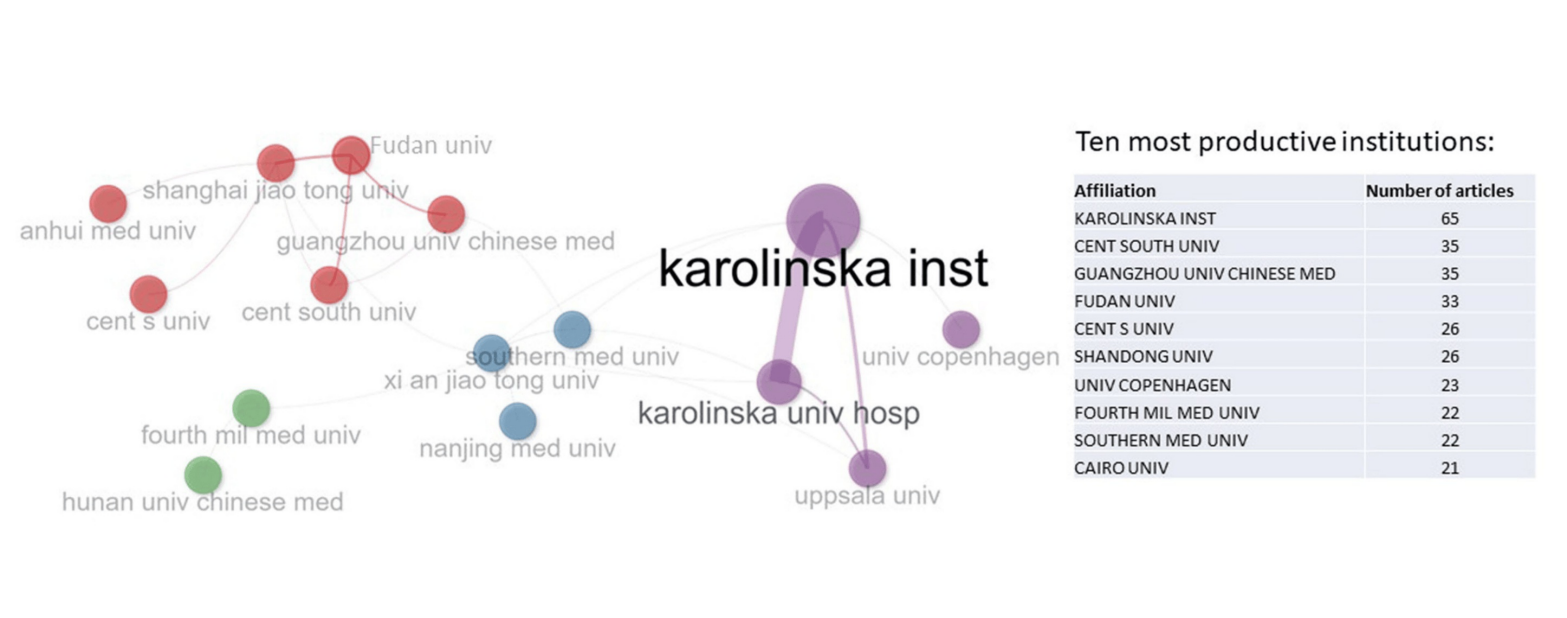
The collaboration network of institutions and the 10 most productive institutions. Karolinska Institute was the leading institution, followed by Central South University, Guangzhou University of Chinese Medicine, and Fudan University.

The collaboration network of the institutions in the study and the top 10 most productive institutions is shown in Figure 4.

Karolinska Institute is the most productive institution, leading the list with 65 articles. Central South University and Guangzhou University of Chinese Medicine both rank second, each with 35 articles.

Journals Analysis

Among the journals in which the analyzed articles were published, Experimental Dermatology (21 articles) and the Journal of Investigative Dermatology (14 articles) stand out. The International Journal of Molecular Sciences ranks third with 11 articles, followed by PLoS One with 10 articles and the European Journal of Dermatology with nine articles. While Experimental Dermatology has the highest number of publications in this field, the journal with the highest local citation count is the Journal of Investigative Dermatology (959 citations), despite having the second-highest number of publications. PLoS One ranks second in citation count with 633 citations, followed by the British Journal of Dermatology with 492 citations. Experimental Dermatology, despite its leading publication count, ranks sixth in citation count with 424 citations. Since 2011, and more prominently in recent years, these leading journals have maintained a relatively steady output of publications in this field.

Co-cited Reference Analysis and Most Influential Articles

The top 10 articles with the highest number of local citations are presented in Table 2.

**Table 2:** Top 10 most locally cited articles (ranked by local citations). LC: local citations; GC: global citations.

Rank	Document	DOI	Title	LCs	GCs	LC/GC ratio (%)	Normalized LCs	Normalized GCs
1	Sonkoly E et al. (2007) [7]	10.1371/journal.pone.0000610	MicroRNAs: novel regulators involved in the pathogenesis of psoriasis?	134	604	22.19	2.00	1.72
2	Zibert JR et al. (2010) [8]	10.1016/j.jdermsci.2010.03.004	MicroRNAs and potential target interactions in psoriasis	84	167	50.30	4.63	2.38
3	Xu N et al. (2011) [9]	10.1038/jid.2011.55	MiR-125b, a microRNA downregulated in psoriasis...	77	175	44.00	3.45	1.86
4	Joyce CE et al. (2011) [10]	10.1093/hmg/ddr331	Deep sequencing of small RNAs from human skin...	76	188	40.43	3.41	1.99
5	Xu N et al. (2013) [11]	10.4049/jimmunol.1202695	MicroRNA-31 is overexpressed in psoriasis...	60	156	38.46	8.71	2.37
6	Meisgen F et al. (2012) [12]	10.1111/j.1600-0625.2012.01462.x	MiR-21 is up-regulated in psoriasis and suppresses T cell apoptosis	58	131	44.27	4.43	02.04
7	Hawkes JE et al. (2016) [13]	10.1038/JID.2015.409	MicroRNAs in psoriasis	55	102	53.92	11.48	2.63
8	Lerman G et al. (2011) [14]	10.1371/journal.pone.0020916	MiRNA expression in psoriatic skin: reciprocal regulation...	51	112	45.54	2.29	1.19
9	Guinea-Viniegra J et al. (2014) [15]	10.1126/scitranslmed.3008089	Targeting miR-21 to treat psoriasis	50	121	41.32	05.09	2.43
10	Yan S et al. (2015) [16]	10.1038/ncomms8652	NF-κB-induced microRNA-31 promotes epidermal hyperplasia...	43	174	24.71	8.11	3.47

The miRNA types mentioned in the titles of these top 10 most locally cited articles include miR-125b, microRNA-31 (in two articles), hsa-miR-99a, and miR-21 (in two articles). The most locally cited article, with 134 citations, is the 2007 research article by Sonkoly E et al. [7], titled "MicroRNAs: novel regulators involved in the pathogenesis of psoriasis?" This article is also the most globally cited, with 604 citations. The second most locally cited article is the 2010 research article by Zibert JR et al. [8], titled "MicroRNAs and potential target interactions in psoriasis."

Keyword Analysis

**Figure 5:**
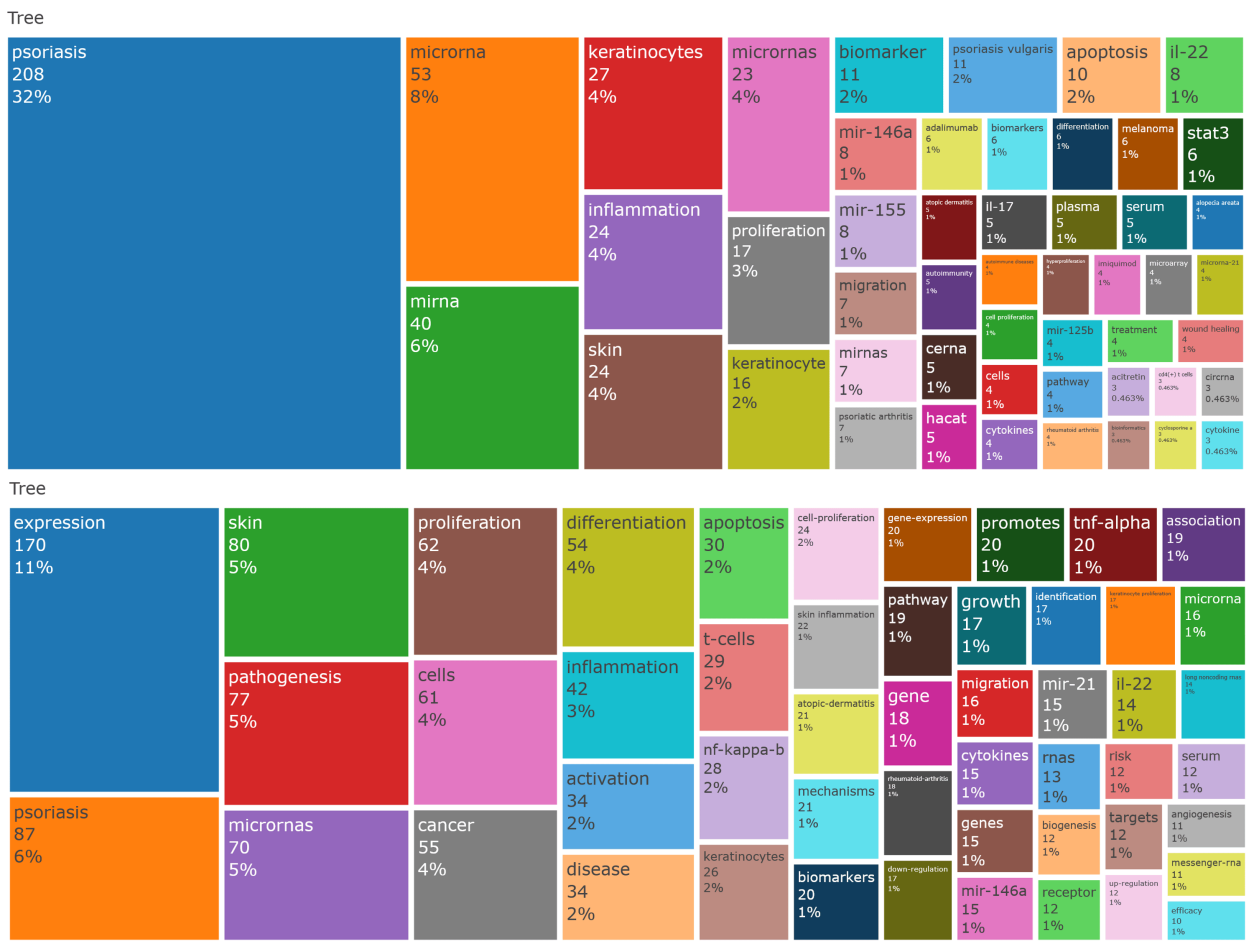
Treemaps illustrating the frequency distribution of research keywords. The upper panel displays the author-provided keywords, where the most frequently used terms were “psoriasis”, “microRNA/miRNA”, “keratinocytes”, and “inflammation”. The lower panel shows the distribution of Keywords Plus terms (indexed keywords), with the most common terms being “expression”, “psoriasis”, “skin”, “pathogenesis”, and “microRNAs”. The size of each rectangle is proportional to the frequency of the keyword.

Figure 5 illustrates the frequency distribution of author-provided and indexed keywords, respectively. The most common author keywords were “psoriasis,” “microRNA/miRNA,” “keratinocytes,” and “inflammation,” while the most frequent Keywords Plus terms were “expression,” “psoriasis,” “skin,” “pathogenesis,” and “microRNAs.”

**Figure 6:**
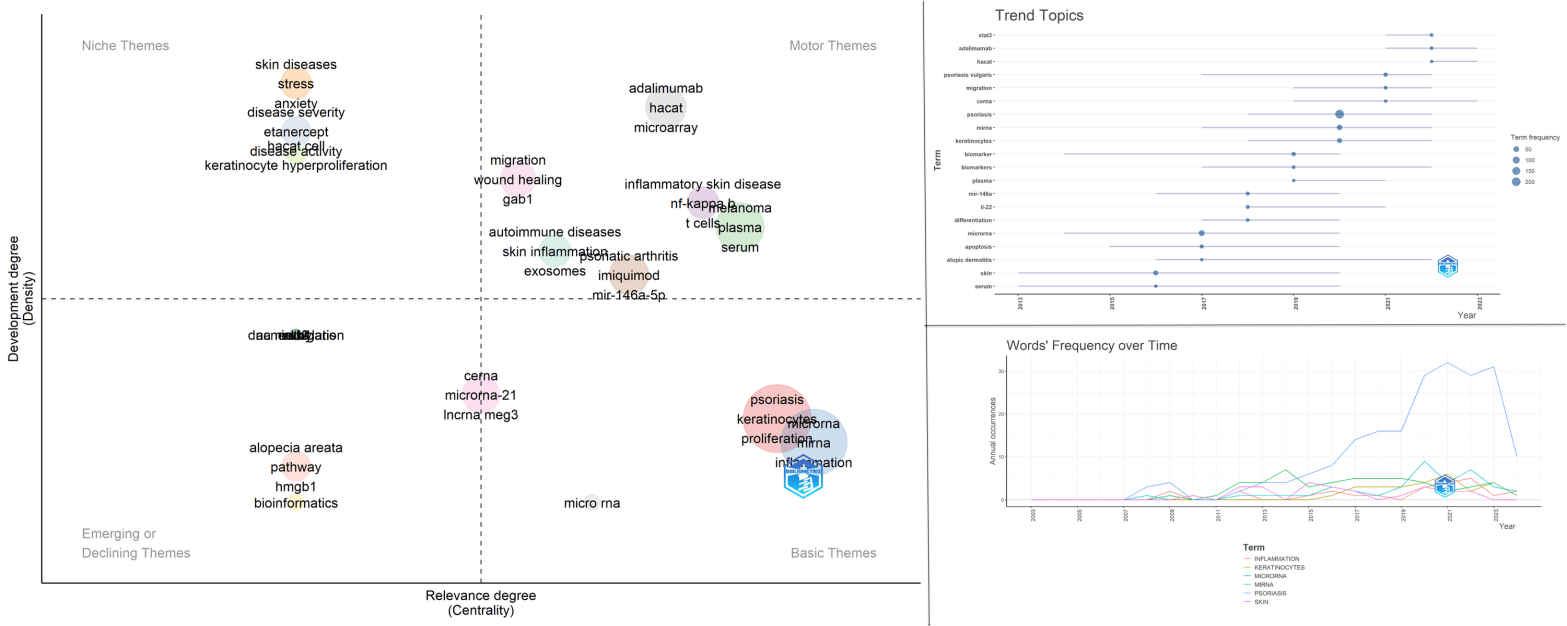
Left: Thematic map of author keywords, categorizing terms into motor, basic, niche, and emerging/declining themes based on density and centrality. Top right: Trend topic timeline showing the emergence of frequently used terms over the years. Bottom right: Temporal frequency of selected keywords, illustrating rising interest in psoriasis-related microRNA (miRNA) topics.

Figure 6 illustrates the thematic map of author keywords (left), the timeline of emerging trend topics (top right), and the temporal frequency of selected keywords (bottom right), demonstrating key research themes, evolving trends, and the increasing emphasis on psoriasis-related miRNA studies.

The five most frequently used author keywords are psoriasis, microRNA, miRNA, keratinocytes, and inflammation, whereas the top five Keywords Plus are expression, psoriasis, skin, pathogenesis, and microRNAs. Thematic mapping of author keywords (left) categorizes terms into motor, basic, niche, and emerging/declining themes. Basic themes include psoriasis, microRNA, miRNA, keratinocytes, inflammation, and proliferation. In the niche themes quadrant, notable terms are disease activity, disease severity, stress, keratinocyte hyperproliferation, and HaCaT cells. The central cluster features ceRNA, microRNA-21, and lncRNA MEG3. Prominent motor themes include miR-146a-5p, HaCaT, skin inflammation, exosomes, and GAB1. The trend topic timeline (top right) highlights recently emerging terms such as STAT3, adalimumab, and HaCaT. The annual frequency graph (bottom right) demonstrates a marked increase in the use of the six most frequent author keywords (psoriasis, microRNA, miRNA, keratinocytes, inflammation, and skin), particularly for psoriasis, with modest increases observed for the others. The most frequently cited psoriasis-related miRNAs and their translational relevance are summarized in Table 3.

**Table 3:** Frequently studied microRNAs (miRNAs) in psoriasis with related pathways, key references, and translational relevance.

miRNA	Dominant pathway/theme	References	Translational tag(s)*	Notes
miR-31	Keratinocyte cytokines (NF-κB/STK40); hyperplasia	Xu N et al. (2013) [11]; Yan S et al. (2015) [16]	biomarker, therapy-interaction	Repeated appearance in top-cited set; maps to STAT3/NF-κB axes
miR-21	T-cell apoptosis; inflammatory signaling	Meisgen F et al. (2012) [12]; Guinea-Viniegra J et al. (2014) [15]	biomarker, therapy-interaction	Targeted in preclinical work; inflammation-proliferation cross-talk
miR-146a	Immune modulation; IL-17 correlation	Hawkes JE et al. (2016) [13]; Sonkoly E et al. (2007) [7]	biomarker	Serum/skin associations; severity correlations reported
miR-203	Keratinocyte function; SOCS-3/STAT3 tie-in	Sonkoly E et al. (2007) [7]	biomarker	Early psoriasis linkage; plausible STAT3 coupling
miR-125b	Keratinocyte proliferation (FGFR2)	Xu N et al. (2011) [9]	biomarker	Recurrent in top-cited set; proliferation-axis

**Figure 7:**
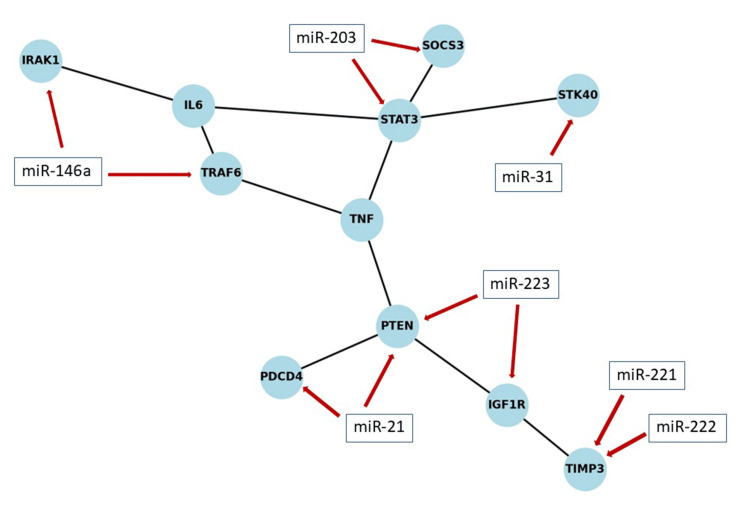
Protein–protein interaction and miRNA–target network based on validated targets of psoriasis-associated miRNAs. miRNA: microRNA.

Figure 7 demonstrates a protein-protein interaction (PPI) network based on validated target genes of psoriasis-associated miRNAs. The constructed PPI network highlights key hubs such as STAT3, PTEN, and TRAF6, where psoriasis-related miRNAs converge on interconnected inflammatory, proliferative, and immune signaling pathways.

Discussion

This analysis reveals a steady and significant growth in miRNA-related psoriasis research over the past two decades, with a marked acceleration following 2007 and peaking in 2021. The observed growth reflects the increasing recognition of miRNAs as pivotal regulators in psoriasis pathogenesis and their promise as diagnostic and therapeutic targets. Citation trends mirrored publication trends, with a slight decline in 2022-2023 likely reflecting shifting research priorities during the COVID-19 pandemic. Notably, highly cited studies frequently centered on key miRNAs such as miR-125b, miR-31, miR-99a, and miR-21.

Key Research Trends

Our analysis showed contributions from 51 countries and 887 institutions, with China, the USA, and Sweden as leading contributors. The Karolinska Institute emerged as the most productive institution, while collaborative patterns revealed increasing international engagement, with 17.6% of articles involving multi-country authorship. Among 2,448 authors, Pivarcsi A and Sonkoly E stood out with the highest local citation counts, reflecting their foundational impact on the field. Publication venues such as Experimental Dermatology and Journal of Investigative Dermatology consistently supported the dissemination of miRNA-psoriasis research, as evidenced by high citation metrics.

Between 2019 and 2023, a notable surge in citations was observed in this field (Figure 2), largely driven by the 2007 study by Sonkoly et al. [7] in PLoS One. This pioneering work, the most cited globally, was the first to link miRNAs to psoriasis, identifying the upregulation of miR-203 and miR-146a in psoriatic plaques. miR-203 was shown to influence keratinocyte function, while miR-146a was linked to immune cells. Highlighting miR-203 as a potential therapeutic target, the study suggested that miRNA-based therapies could outperform single-protein treatments, significantly shaping subsequent research trends.

The 2010 study by Zibert et al. [8], titled "MicroRNAs and potential target interactions in psoriasis," examined miRNA-mRNA interactions in psoriatic and healthy skin. It identified 42 upregulated and five downregulated miRNAs in psoriatic lesions. Among these, the overexpression of miR-221 and miR-222 in keratinocytes was shown to degrade TIMP3, a key interaction in psoriasis pathogenesis. The authors suggested that miRNAs function as fine-tuners in the molecular framework of psoriasis and represent potential therapeutic targets. To illustrate the biological relevance of top-cited miRNAs, we constructed a STRING-based protein-protein interaction network using validated targets. Key nodes such as STAT3, TRAF6, and PTEN emerged, showing convergence on inflammatory signaling, keratinocyte proliferation, and immune regulation, which represent core pathogenic mechanisms in psoriasis.

In the graph depicting the annual usage frequency of the authors’ keywords, it is observed that the six most frequently used authors’ keywords (psoriasis, microRNA, miRNA, keratinocytes, inflammation, and skin) have shown a noticeable increase in usage in recent years, particularly for psoriasis, with a moderate increase for the others. In the thematic map, the basic themes, which are the most frequently occurring words, are primarily "psoriasis," "microRNA," "miRNA," "keratinocytes," "inflammation," and "proliferation." Psoriasis is characterized by the hyperproliferation and abnormal differentiation of keratinocytes, as well as the abnormal production of inflammatory cytokines and chemokines. The presence of the words "keratinocytes," "proliferation," and "inflammation" among the basic themes suggests that the articles are focused on elucidating the roles of miRNAs in fundamental mechanisms of psoriasis pathogenesis, such as inflammation and keratinocyte proliferation. Among the extensively studied miRNAs in psoriasis, miR-21 and miR-222 have been found to affect cell growth and apoptosis in keratinocytes. MiR-31, another frequently studied miRNA, has been shown to regulate the production of inflammatory cytokines, including IL-1β, CXCL1/5/8, and IL-8, by targeting STK40, a negative regulator of NF-kB [8,11]. Similarly, miR-223 has been shown to be expressed in Th17 cells and to enhance the inflammatory response by increasing IL-17 production [4]. A study by Ichihara et al. demonstrated that decreased expression of miR-424 and the consequent upregulation of mitogen-activated protein kinase kinase 1 (MEK1) and cyclin E1 led to keratinocyte proliferation [17].

In the thematic map, the central section features key terms such as "ceRNA," "microRNA-21," "lncRNA," and "MEG3" (maternally expressed gene 3) that are considered important but under-researched themes. The presence of ceRNA, lncRNA, and MEG3, alongside microRNA-21, in the central area of the thematic map highlights the significance of noncoding RNA studies in psoriasis. This indicates that these themes are crucial for researchers considering studying the pathogenesis and treatment of psoriasis. MicroRNA-21 is a prominent type of miRNA in psoriasis research; however, its central position on the graph may indicate that further research is still needed.

In the motor themes area, the prominence of the terms "mir-146a-5p," "HaCaT," "skin inflammation," "exosomes," and "GAB1" (GRB2-associated binding protein 1) indicates that research in this field is concentrated on these themes. The presence of these terms in the thematic map provides valuable guidance for researchers aiming to better understand the pathogenesis of psoriasis and to develop potential therapeutic strategies.

Translational Implications and Clinical Relevance

In the trend topic of the authors' keywords graph, we observe that "stat3," "adalimumab," and "HaCaT" have emerged prominently in recent years. STAT3, when activated, supports keratinocyte proliferation and survival. miR-203 targets SOCS-3 (suppressor of cytokine signaling 3), an inhibitor of STAT3. Downregulation of SOCS-3 leads to the activation of STAT3, thereby enhancing inflammatory responses and keratinocyte proliferation [8]. Other miRNAs, such as miR-21 and miR-146a, are also upregulated in psoriasis and indirectly influence the STAT3 pathway by modulating pro-inflammatory cytokines like IL-6 [7,13,15].

In the niche themes area, "disease activity," "disease severity," "stress," "keratinocyte hyperproliferation," and "HaCaT cells" are prominent themes. The use of HaCaT cells in psoriasis-related studies, due to their ability to model psoriasis pathology, can facilitate the rapid advancement of research aimed at elucidating the molecular mechanisms and specific targets of psoriasis-associated miRNAs [18].

In psoriasis, dysregulated miRNAs can be detected in peripheral blood and/or affected psoriatic skin [13]. The relationship between various miRNAs and the severity of psoriasis has been investigated, with reports indicating a positive correlation between serum levels of certain miRNAs and disease severity [19,20]. Hawkes et al. [13] found that miR-146a, one of the frequently studied miRNAs in psoriasis, shows a positive correlation with the Psoriasis Area and Severity Index (PASI) and serum IL-17 levels. Similarly, another study found that miR-223 and miR-143 were highly expressed in peripheral blood mononuclear cells (PBMCs) of psoriasis patients compared to healthy controls and showed a significant correlation with PASI scores. In this latter study, the authors also demonstrated that miR-223 and miR-143 in PBMCs were significantly downregulated following methotrexate treatment, which coincided with a reduction in psoriasis lesions in patients [21]. The increased expression of miRNAs with disease severity suggests their significant role in the pathogenesis and potentially the progression of psoriasis. However, some studies have not observed the expected relationship between miRNA alterations and disease severity [22].

The inclusion of treatment agents like "adalimumab" in the thematic map indicates that miRNAs are studied not only in the context of pathogenesis but also for therapeutic research. A study reported that etanercept treatment significantly reduced the serum levels of miR-106b, miR-26b, miR-142-3p, miR-223, and miR-126 [23]. In the future, changes in miRNA levels following biologic drugs might be used to monitor the efficacy of this treatment. The high conservation and stability of circulating miRNAs, along with their easy detection in blood samples, make them attractive potential biomarkers [3]. Our findings suggest that current literature on miRNAs in psoriasis covers both their roles in preclinical models and their potential as biomarkers and therapeutic targets. This reflects a shift from basic discovery to more clinically focused research, highlighting the growing translational relevance of miRNA studies in psoriasis.

Limitations

Our study has several limitations. First, we only used data from the Web of Science Core Collection database, as it offers the most comprehensive and standardized dataset for bibliometric analyses. Second, we included only literature written in English to ensure uniformity and minimize inconsistencies in data extraction and interpretation.

## Conclusions

In conclusion, this study presents the first comprehensive scientometric mapping of miRNA research in psoriasis. The field demonstrates growing international collaboration, high citation impact, and a clear shift from descriptive studies toward mechanistic and translational research. These findings provide a structured overview that may guide future research and support the prioritization of candidate miRNAs for diagnostic panels and therapeutic monitoring in psoriasis.
